# Hypercalcemia-Leukocytosis syndrome and adenosquamous lung carcinoma: An overlooked conjugation

**DOI:** 10.32604/or.2022.023450

**Published:** 2022-07-13

**Authors:** JOÃO OLIVEIRA PEREIRA, JOÃO GAMA, DIANA FERREIRA, ARSÉNIO SANTOS

**Affiliations:** 1 Pulmonology Department, Coimbra Hospital and University Centre, Coimbra, 3004-561, Portugal; 2 Faculty of Medicine of the University of Coimbra, Coimbra, 3000-370, Portugal; 3 Anatomical Pathology Department, Coimbra Hospital and University Centre, Coimbra, 3004-561, Portugal; 4 Internal Medicine Department, Coimbra Hospital and University Centre, Coimbra, 3004-561, Portugal

**Keywords:** Hypercalcaemia-leukocytosis syndrome, adenosquamous lung carcinoma, skull metastases, percutaneous lymph node biopsy

## Abstract

Hypercalcaemia and leukocytosis are two paraneoplastic conditions associated with poor prognosis. Adenosquamous carcinoma is a rare and aggressive histological subtype of lung cancer consisting of adenocarcinoma and squamous cell components. We report the case of a 57-year-old male smoker who was admitted to the Emergency Room with skull and neck tumefactions, confusion and deteriorated general condition. The complementary study in the ER revealed severe hypercalcaemia (19.8 mg/dL), leukocytosis (18.7 × 10^9^/L) and extensive osteolytic lesions of the skull on cranioencephalic computer tomography (CT). The patient was stabilized and admitted. Thoracoabdominopelvic CT showed lung parenchyma consolidation with necrotic areas, supra and infradiaphragmatic adenopathies and scattered osteolytic lesions. Percutaneous lymph node biopsy was consistent with metastasis of adenosquamous lung carcinoma. The patients’ clinical situation evolved unfavourably after hospital-acquired infection. This case is characterized by a rare presentation of advanced stage adenosquamous lung carcinoma with scattered osteolytic lesions and severe hypercalcaemia-leukocytosis syndrome, an underrecognized marker of poor prognosis.

## Introduction

Hypercalcaemia and leucocytosis are two frequent paraneoplastic syndromes of debatable aetiology and are particularly common in patients with lung cancer [1,2]. These conditions correlate with poor prognosis and rarely occur concomitantly [1–3].

Adenosquamous lung carcinoma is a mixed, rare and aggressive histological subtype of lung cancer, characterized by the presence of adenocarcinoma and squamous cell carcinoma components [4].

With the report of a rare case of advanced lung cancer with severe hypercalcaemia of malignancy, paraneoplastic leucocytosis and extensive osteolytic lesions, we intend to conduct a brief review of the literature on these conditions and to raise awareness on the prognostic significance of the aforementioned biomarkers.

## Case Report

57-year-old-male, sent to the Emergency Room due to altered level of consciousness and mental confusion in the previous 3 days. He was dependent for daily activities and bedridden for the previous week. He had presented complaints of anorexia, weight loss, and swelling of the skull with six months of progression.

His previous medical history included active hepatitis C virus infection, chronic alcoholism, and active smoking, with an apparently high exposure which could not be quantified. He was taking alprazolam and olanzapine.

In the ER, he was conscious and disoriented (Glasgow Coma Scale of 13 points). He was apyretic, slightly tachycardic (101 bpm), normotensive and had a peripheral oxygen saturation of 92%.

Head and neck inspection revealed poor oral hygiene, dehydration and subcutaneous thickening of the skull base without defined masses. Occipital and parietal soft tumefactions and anterior cervical petrous lymph nodes could be felt on palpation. The remaining objective examination, although limited by non-cooperation, showed no alterations.

Analytically, we identified acute kidney injury associated with hypercalcaemia, hypernatraemia and hypokalaemia (creatinine 2.06 mg/dL (1.72–1.18 mg/dL), calcium 19.8 mg/dL (8.8–10.6 mg/dL), ionized calcium 8 mg/dL (4.5–5.3 mg/dL), albumin 3.5 g/dL (3.5–5.2 g/dL), sodium 149 mmol/L (136–146 mmol/L), potassium 3.0 mmol/L (3.5–5.1 mmol/L)), as well as elevation of C-reactive protein (7.91 mg/dL (0–0.5 mg/dL)) and leukocytosis (18.7 × 109/L (4.0–10.0 × 109/L)) with neutrophilia, the latter persisting despite antibiotic therapy. Procalcitonin and blood, urine and sputum cultures were negative on admission.

**Figure 1:**
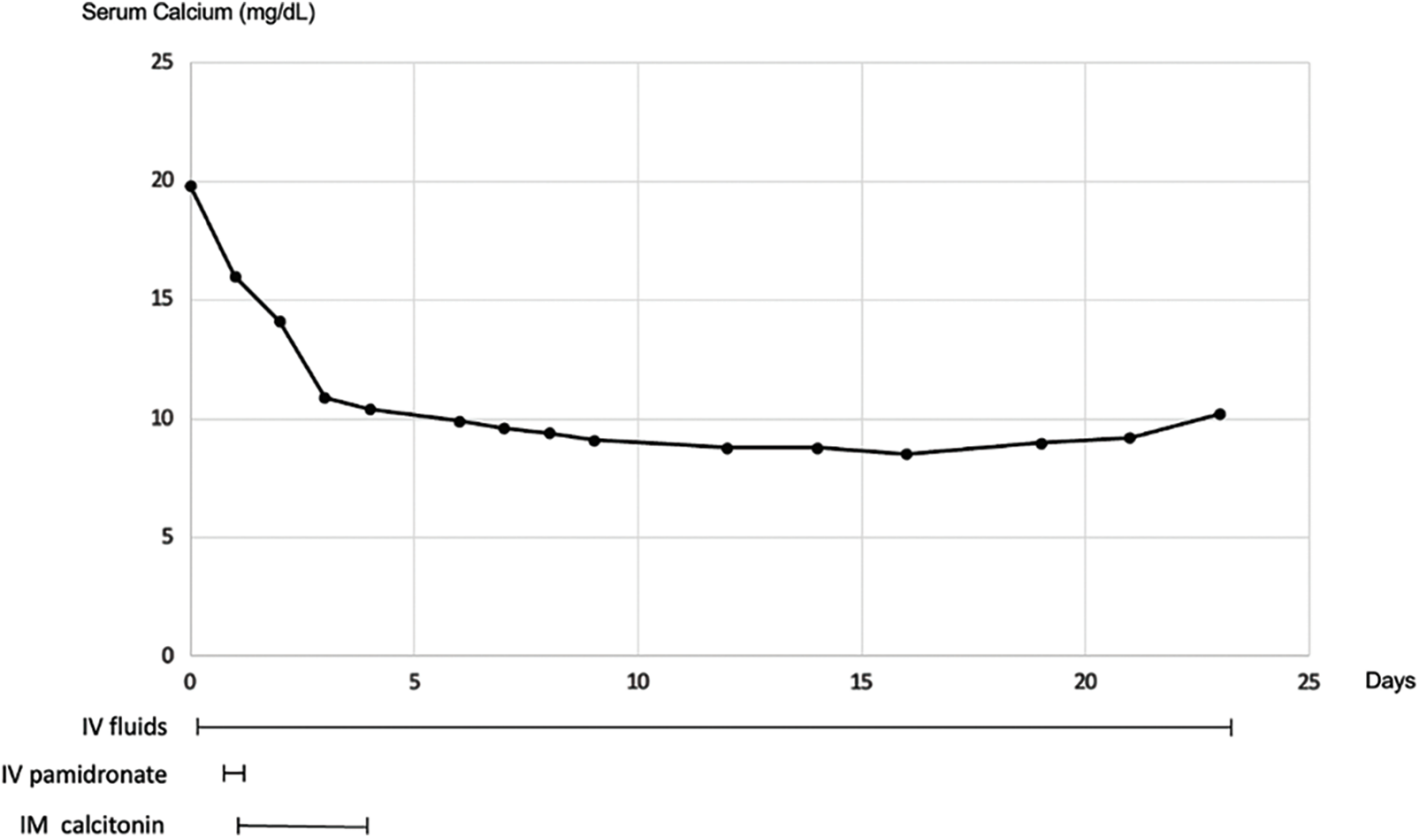
Severe hypercalcaemia (19.8 mg/dL) was corrected with intravenous (IV) fluid therapy, IV sodium pamidronate (90 mg, single dose, on day 1) and intramuscular (IM) salmon calcitonin (300 I.U. q12 h, on days 1–3). Normocalcaemia was achieved on day 4.

We initiated treatment for severe hypercalcaemia and hypokalaemia with IV fluid therapy, IV sodium pamidronate (90 mg, single dose, on day 1) and intramuscular salmon calcitonin (300 I.U., q12 h, on days 1–3) and admitted the patient to the ward after calcium normalization (day 4) (Fig. 1).

Complementary study of hypercalcaemia showed normal parathyroid hormone (21 pg/mL (9–72 pg/mL)) and severely low levels of 25-hydroxyvitamin D (9.3 ng/mL), with normal phosphate and low magnesium levels (1.7 mg/dL (1.8–2.6 mg/dL)). PTH was reassessed at 35 pg/mL (day 5).

**Figure 2:**
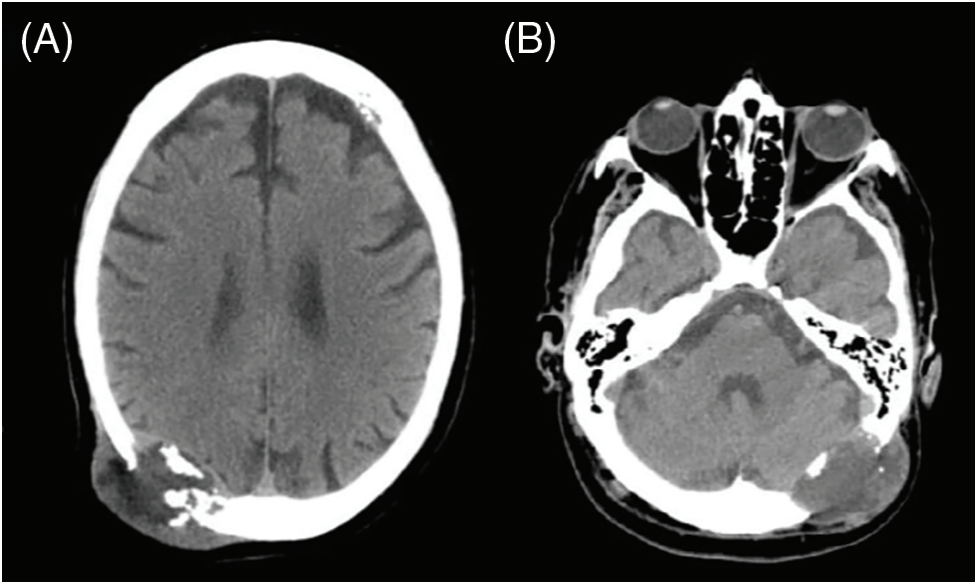
Head CT scan revealed extensive osteolytic lesions, the largest in the right parietal (A) and left occipital (B) regions.

Head CT demonstrated extensive heterogeneous osteolytic lesions of the skull, conditioning deviation and moulding of the cerebral parenchyma and rectification of the cerebellous hemisphere (Figs. 2A and 2B).

**Figure 3:**
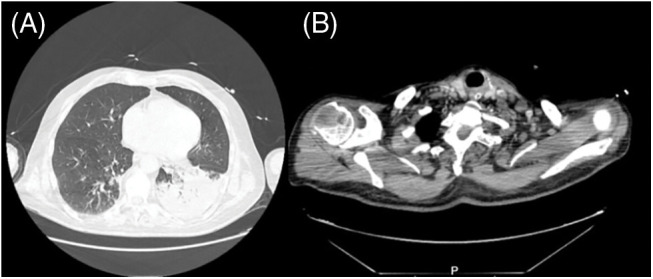
Thoracicabdominopelvic CT scan revealed extensive consolidation to the lower lobe of the left lung with foci of necrosis (A) and presumably metastatic lesions to lymph nodes, peritoneal cavity and bones (B).

Abdominal and renal ultrasound revealed heterogenous liver texture suggestive of chronic hepatopathy. Genotype 1a HCV infection with a viral load of 2992 IU/mL (log 3.4) was identified. We continued the study with cervicothoracoabdominopelvic contrast-enhanced CT (Figs. 3A and 3B), which revealed an extensive area of consolidation to the lower lobe of the left lung and cervical, supraclavicular, axillary and mediastinal adenopathies, the latter with hypodense centre probably reflecting necrosis. Several peritoneal nodules, as well as lytic lesions affecting the 10th thoracic vertebra, right tenth rib, right sacral wing, right iliac wing and the head of the right humerus were also noted. The panel of tumour markers revealed elevated CEA (11 ng/mL (<5.4 ng/mL)), CYFRA 21.1 (13 ng/mL (<3.3 ng/mL)), NSE (26 ng/mL (<15 ng/mL)) and SCC (11 ng/mL (<1.5 ng/mL)).

**Figure 4:**
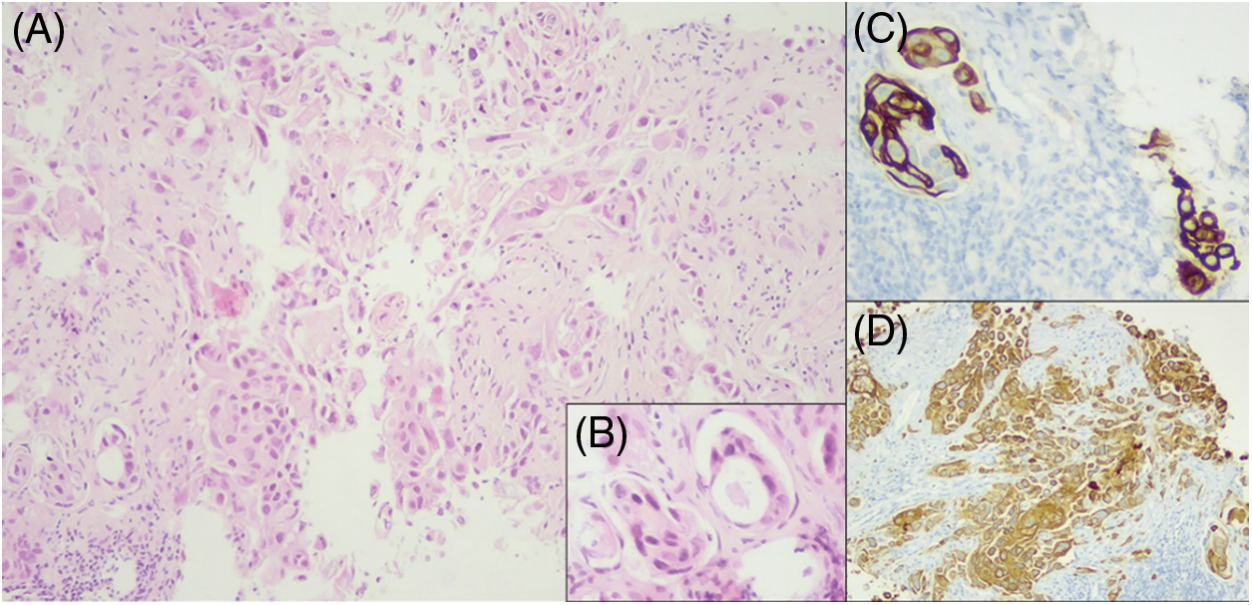
Cell block of the cervical lymph node biopsy. (A) Neoplasm with solid and focal acinar pattern. Both components are composed of cells with eosinophilic cytoplasm, increased nucleus-cytoplasm ratio, pleomorphic nuclei and prominent nucleoli, HE 100× magnification. (B) Acinar component of the neoplasm, HE 400× magnification. (C) Immunohistochemistry for CK7, highlighting the acinar differentiation, 400× magnification. (D) Immunohistochemistry for CK5/6, highlighting the epidermoid component, 100× magnification.

We performed fine needle aspiration biopsy of a cervical lymph node and identified a mixed, predominantly solid neoplasm with an epidermoid component with immunoexpression for CK5/6 and glandular component with CK7 expression. Despite being negative for TTF1, the sample was compatible with adenosquamous cell carcinoma of the lung (Fig. 4).

Due to the poor prognosis associated with this plurimetastatic tumour and respective para neoplastic syndrome, we started palliative care. The patient later contracted nosocomial infection, with respiratory failure and marked level of consciousness alteration (GCS 3 to 6) and died during hospitalization, despite calcaemia normalization.

## Discussion

This case is characterized by a rare presentation of advanced-stage adenosquamous cell carcinoma of the lung, with hypercalcaemia-leukocytosis syndrome and scattered osteolytic lesions.

This histological subtype represents only 0.4 to 6.5% of lung carcinomas in surgical series [5,6], and is associated with relatively nonspecific symptoms at presentation [7]. It is histologically characterized by its complexity, with the presence of adenocarcinoma and squamous cell carcinoma components, each in proportions >10% of the surgically resected lesion [4]. The existing studies are based on surgical specimens as sampling limitation of incisional biopsies usually hampers the diagnosis through this technique.

The complexity, low incidence and difficulty of diagnosing the disease are major obstacles to its study. Its histogenesis remains unclear and there is no consensual standardized treatment, which is currently based on guidelines for non-small cell lung cancer [8]. These facts have added relevance considering its aggressiveness, with an overall survival significantly lower than that of adenocarcinoma and squamous cell lung carcinomas [9], in addition to evidence of earlier lymph node metastasis [10].

Hypercalcaemia is relatively common in patients with cancer, lung carcinomas being among the most common [11]. Its prevalence among the latter may reach 25% and portends poor prognosis [3,12]. In a great proportion of patients with hypercalcemia of malignancy, the diagnosis of cancer is not difficult to achieve [13]. From the pathophysiological point of view, four major mechanisms, in order of prevalence, may explain hypercalcaemia [14,15]:

– Tumour secretion of parathyroid hormone-related protein (PTHrP), in about 80% of all cases;
– Osteolytic metastases with local release of cytokines (including osteoclast activating factors), in 20% of all cases;
– Tumour production of 1,25-dihydroxyvitamin D (calcitriol);
– PTH secretion (either primary or ectopic).

Thus, after ensuring proper patient hydration and excluding pseudohypercalcaemia (by quantifying serum protein levels), PTH should be measured. These molecules have resembling biochemical structure and overlapping effects on calcium homeostasis, one of the few exceptions being the regulation of calcitriol levels [13,16]. In the presence of non-PTH-mediated hypercalcemia, PTH levels should be suppressed or low-normal. If severe hypercalcemia is present, non-suppressed PTH could point towards rarely described ectopic tumoural secretion of PTH [17] or even primary hyperparathyroidism, a condition with increased prevalence among patients with cancer [18]. In the suspicion of non-PTH-mediated hypercalcemia, PTHrP and vitamin D metabolites (including 25-hydroxyvitamin D and/or 1,25-dihydroxyvitamin D levels) should be dosed. PTHrP elevated levels are associated with humoral hypercalcaemia of malignancy. Its dosing, however, may be omitted in cases where the diagnosis of cancer is established or evident (e.g., advanced squamous cell carcinomas of the lung) [13]. On the other hand, the elevation of vitamin D metabolites should raise suspicion of lymphoma or other granulomatous disease. Osteolytic lesions, including multiple myeloma and solid tumour metastases, are to be considered as a cause of hypercalcaemia, particular, but not exclusively, in the presence of normal PTHrP. PTH and vitamin D metabolite levels.

Our patient had severe hypercalcaemia, low-normal PTH and severely low 25-hydroxyvitamin D levels. Due to unavailability, we could not measure serum PTHrP levels. In the presence of severe hypercalcemia, low-normal (non-suppressed). PTH could point towards inappropriate secretion of PTH, which mighthelp explain the severity of our patient’s hypercalcaemia. It should be noted, however, that there is discussion around PTH lower cut off and as to when primary hyperparathyroidism may be ruled out, as PTH reference range is influenced by the extent of vitamin D repletion [19] and renal function [20] (both abnormal in our patient) Besides, on the study by Hiraki et al. [21], all patients with lung cancer and hypercalcaemia of malignancy in whom serum PTH was measured had levels within the normal range. Ectopic PTH secretion could not be further ruled out in our patient given his clinical status. However, normocalcaemia and normal PTH levels were successfully achieved under combined therapy with IV fluids, pamidronate and salmon calcitonin. While bisphosphonate might have a direct anti-tumour effect [22], resistance to this treatment, possibly related with PTHrP secretion [23], has been described in some lung cancers, and may be addressed with calcitonin [24]. We believe PTHrP might have had a significant role in our patient’s calcaemia, even though we were unable to either confirm or exclude it. The presence of scattered osteolytic lesions cannot, however, be overlooked and has probably contributedwith tumour-stimulated, osteoclast-mediated bone destruction [25].

Leukocytosis has a high prevalence among patients with lung cancer (16%–30%) and is also a known marker of poor outcome [3,25]. In our case, differential mechanisms included, in order of likelihood, infection, malignancy, smoking, reactive causes and drugs [26]. Completely ruling out prior reactive causes was not feasible due to the patient’s level of consciousness, but no prescription of leukocytosis-associated drugs was registered. Negative blood, urine and sputum cultures, as well as persistence after antibiotic therapy, smoking cessation and controlled drug administration mitigated the probability of infection, smoking and medication, as causes, respectively. On the other hand, malignant processes, as other injuries responsible for cell damage and death, are known to be associated with the release of necrotic debris and several other inflammatory factors, especially in the presence of high tumoural activity [27,28]. In the absence of any other ascertained cause, this was the most probable etiology of leukocytosis in our patient.

Finally, concomitant hypercalcaemia and leukocytosis, forming a paraneoplastic syndrome of hypercalcaemia-leukocytosis, was first pointed in tumours of the oral cavity [29] and later confirmed in lung tumours [4]. Its occurrence has also been described in other types of cancer, including carcinoma of the skin [30], bone [31], penis [32], urothelium [33], endometrium [34], tongue [35] and cholangiocarcinoma [36]. Its incidence is lower (0.5% of lung neoplasms at presentation) and associated with worse prognosis than isolated hypercalcaemia but not different from leukocytosis alone [3]. The subjacent mechanism is matter of debate, but may include the simultaneous production of granulocyte colony-stimulating factor (G-CSF) and PTHrP by neoplastic cells [35], or G-CSF alone, by stimulation of both haematopoietic progenitors and osteoclasts [3].

The only case series documenting hypercalcaemia-leukocytosis syndrome in lung cancer we found [3] included 1149 patients with lung cancer, among whom 14 (1.22%) had adenosquamous cell carcinoma and only 6 patients (0.52%) presented hypercalcaemia-leukocytosis syndrome. One single patient was diagnosed with both conditions.

## Conclusion

With this report we intend to describe a case of severely high calcaemia associated with lung adenosquamous carcinoma and to raise awareness on hypercalcaemia-leukocytosis syndrome as a likely underreported and underused marker of poor prognosis.

## Data Availability

No datasets were generated or analyzed during the current study. Anonymized patient information may be consulted by contacting the correspondent author.
